# Examining Opportunities, Challenges and Quality of Life in International Retirement Migration

**DOI:** 10.3390/ijerph182212093

**Published:** 2021-11-18

**Authors:** Yuan Tang, Tara Rava Zolnikov

**Affiliations:** 1 School of Behavioral Sciences, California Southern University, 3330 Harbor Blvd, Costa Mesa, CA 92626, USA; mary_tang@yahoo.com; 2 Department of Community Health, National University, 9393 Lightwave Ave., San Diego, CA 92123, USA

**Keywords:** amenity migration, lifestyle migration, retirement migration, climate migration, retirement abroad, long-stay tourism, economic migration

## Abstract

As the world has become more interconnected due to the invention and innovation of communication and transportation technologies, more individuals than ever before have been able to travel long distances. In recent years, a growing number of physically able adults in late adulthood have chosen to move across national borders to less costly countries in order to obtain better quality of life upon reaching retirement age. In light of this under-researched but increasingly popular retirement trajectory, this research aimed to provide more insight into the opportunities and challenges that international retired migrants have encountered while retiring abroad. Through the lens of humanistic theory, this research employed a systematic review of research literature, the majority of which were peer-reviewed studies published within the last five years. The reviewed studies (n = 22) conducted spanned four out of seven continents, with heavy emphasis on Europe, the Americas, and Asia. Research results indicated that many of the international retired migrants took advantage of the opportunities of pleasant weather, lower cost of living, and various amenities offered by their host countries to enhance their quality of life by engaging in an active and meaningful lifestyle. However, language barriers, lack of social support, rising healthcare costs, increases in the cost of living, uncertain political climate, and different healthcare practices in their host countries, presented considerable challenges to many international retirees.

## 1. Introduction

Retirement, which marks the end of one’s working career, is an important phase in an individual’s life. In the last few decades, more Americans have lived longer and healthier lives as a result of medical advances. According to the U.S. Census Bureau’s 2017 National Population Projections, by 2035, there will be more people 65 years and older than people under the age of 18; roughly 1 in every 5 residents will be of retirement age [1]. Based on research conducted by the Pew Research Center, it is estimated that 10,000 Americans will turn 65 every day for the next 20 years [2]. While more Americans are heading into retirement age, an emerging trend of retirees moving to lower-cost countries for a higher standard of living has been gaining increased media attention. Research has found that the Baby Boomer generation—individuals born between 1946 and 1964—are better educated and show more vitality and longevity than their predecessors [3]. They perceive retirement as a life phase giving them the opportunity to explore new ground, rather than as a period of rest and relaxation, as the previous generation viewed retirement [4]. According to ethnographic research among diverse retirees in North America and Europe, for decades Baby Boomers in North America and Europe have prized personal freedom and independence, and they are now carrying those values into retirement [5]. Some Baby Boomer retirees have chosen to retire abroad in response to insufficient retirement savings, a fear of rising medical costs, and a lack of faith in how adequately the government and state services would take care of them [5].

Guided by PRISMA (Systematic Reviews and Meta-Analyses) protocol, this study engaged in extensive search, review, and analysis of already published studies on international retirement migration (IRM) and integrated findings to identify key themes, perspectives, and meanings that have been central to retirees or soon-to-be retirees. Humanistic theory, which is essential to elucidate an individual’s motivation, meaning-making, and personal agency [6], was used as the theoretical framework to conceptualize the IRM phenomenon. Thus, stakeholders and psychotherapists can draw inferences that are conducive to their respective decision-making and clinical practice.

## 2. Materials and Methods

With increased globalization, and advanced telecommunication and transportation technologies, there have been a growing number of individuals, mostly from the developed countries, who have retired or have been in the process of planning to retire across national borders to the developing countries, seeking better quality of life in their retirement. The emerging trend of IRM has become a multi-causal aspirations, multi-destinations, and multi-pathways phenomenon that has social, cultural, economic, spatial, environmental, political, demographical, and legal implications for both sending and receiving countries. Despite its growing popularity, IRM has been largely invisible in academia, in public discourse, and in debates on immigration policy. Thus, a systematic review was used to bridge the research gap by an extensive search, review, and analysis of already published studies on IRM in order to identify key themes, perspectives, and meanings that have been central to retirees or soon-to-be retirees.

**Figure 1:**
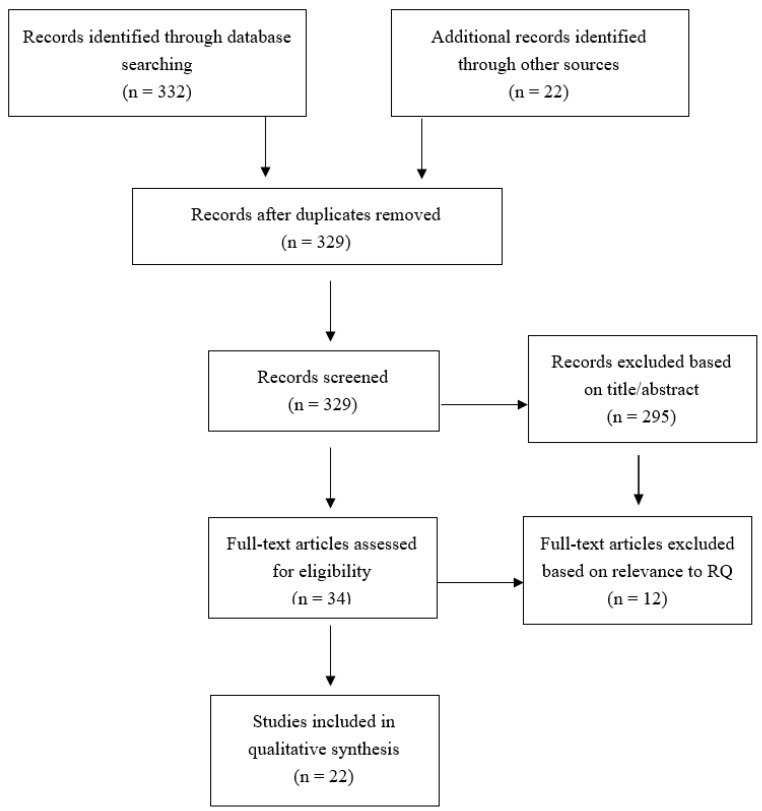
Systematic review PRISMA flow diagram.

The paper only included articles research articles addressed clearly focused research interests, including the motivation and constraints of retiring abroad, the adjustment after becoming settled into the new home countries, the healthcare needs of the international retired migrants as well as the provision of healthcare by the receiving countries, and the counseling services provided to the international retirees and other stakeholders. PRISMA protocol was used n facilitating the appraisal of the published literature and to guide the data collection process. (Figure 1).

A total of 329 research articles pertaining to the IRM phenomenon were selected. Among the articles selected, 22 articles were chosen following the PRISMA guidelines (Table 1). The majority of which were peer-reviewed articles that had been published within the last five years, to ensure their credibility and scientific merit. Articles older than five years were permitted to be included if they bridged a gap of deficiencies in the available research.

IRM historically has been associated with amenity or lifestyle migration among wealthy people who have moved, permanently or temporarily, to other countries for a better quality of life. With increased globalization and advanced telecommunication and transportation technologies, IRM has no longer remained the preserve of an affluent population who can travel long distances and migrate abroad. Despite these socioeconomic differences, it is of increased interest to understand what contributes to aging adults to leave familiar home countries and relocate to foreign destinations that offer changes in culture, lifestyle, and customs. These stark changes can adversely affect the retired migrants’ sense of well-being and increase their vulnerability as they lose their familiar cultural norms and social support after settled in their new host countries.

### 2.1. Factors Associated with Retiring Abroad

With the increase in longevity, global population aging has become one of the most critical demographic features in the 21st century. Aging successfully is associated with several aspects (e.g., social connections, physical health, cognitive capacity, etc.), but these aspects can often be stifled by financial constraints [7]. As a result, many retirees have chosen to retire abroad in response to inadequate retirement savings, rising medical costs and cost of living, social security uncertainty, and a shortage of skilled eldercare health workers [5,8,9]. Additionally, this cohort of generational retirees are distinguished from earlier generations by the relationship with technology (e.g., more adapt to new technology), education attainment (e.g., more educated), and attitude toward career and organizations (e.g., choose early retirement) [5,7,9,10]. That said, these retirees also have shown more vitality and longevity than their predecessors [4,11]. For decades, this generation have prized personal freedom and independence, and they have taken responsibility for their own wellbeing [5]. Unsurprisingly, as they have transitioned out of the labor force, they have carried those values into retirement [5]. Many of them have embraced the idea of successful aging and have carefully crafted where and how they planned to age [5,12]. With the invention and innovation of communication and transportation technologies, many individuals have already traveled abroad and even have had prior experiences living abroad. The Internet has made it easy to network with other expatriates who currently were living abroad, and to access mass media which promoted international living (e.g., International Living Magazine and website).

Marrow and von Koppenfels [13] discovered facilitators that aspire individuals to migrate; these factors include the motivation to minimize one’s personal financial risk, the degree of one’s political ideology and a sense of belonging to the U.S, the level of one’s cultural capital such as linguistic ability (ability to speak at least one foreign language), and whether a retiree had prior travel/tourism experience, social networks with prior migrants, and Internet access to websites which advertised retirement abroad. Using Pickering and colleagues’ [14] categorization scheme, the pull factors by which the positive aspects of a foreign country have attracted international migrants to move and retire there (Table 2).

### 2.2. Retirees’ Experiences with Retiring Abroad

The common factors that have influenced individuals’ decisions to retire abroad included the opportunity to enjoy a warm, pleasant climate which permitted outdoor activities, to explore the cultural and natural attractions which the country offered, and to take advantage of the relatively inexpensive cost of living. These factors have often been associated with improved health, a slower pace of life, and an active social involvement [5,15,16,17]. Many of the international retired migrants became landowners or business owners (e.g., restaurant, tourism, retirement home, etc.) and participated in the community by volunteering in local charities, teaching English, or holding positions of responsibility in community groups and activities [5,8,15,18,19,20,21]. However, there have been several obstacles and disadvantages that retired migrants experienced after settling in new home countries. The language barrier has been the greatest difficulty for retirees who are unable to communicate using the local language in new home countries [18,22,23]. The ability to speak the local language is essential to communicate an individual’s basic needs and desires. When migrants are unable to speak the local language in host countries, the language barrier limits migrants’ abilities to interact with locals in any meaningful way, and therefore negatively affects social and cultural adaptation and integration in host countries [16,20]. Furthermore, the language barrier impeded migrants’ access to healthcare and services, which can result in retiree’s decision to repatriate to their country of origin to receive medical care [22,23]. Additionally, cultural differences between the retired migrants and caregivers from a host country were also viewed as challenging in determining how care needs were assessed and how medical conditions were diagnosed as well as how the subsequent decision-making and treatment approach were made and delivered [8]. Finally, dissatisfaction, loneliness, and frustration often emerged as acculturative stress that negatively affected the retirees’ quality of life when the retired migrants were unable to adjust themselves to host countries’ culture or were disappointed that host countries did not uphold expectations. There were many push factors for unfavorable or detrimental features of the foreign destination that compelled the international retired migrants to leave (Table 3).

### 2.3. Effects of International Retirement Migration

Population aging has generated significant challenges in today’s societies. Many individuals in high-income countries on fixed incomes with inadequate retirement savings have sought an alternative retirement life in low-income countries, wherein more of a middle-class lifestyle can be afforded and achieved. The considerable differentials in cost of living and wages between the sending and receiving countries have been the most essential contextual factors that have propelled retirees’ transnational mobility and the development of the transnational retirement industry [8,9]. Transnational retirement has become desirable to many retirees in low-income countries because of the existence of global imbalances in wealth and privilege between the developing countries and the developed countries [9,24,25,26].

Had there been no wealth differentials, transnational retirement industry may not exist at all. This type of movement not only affects the person migrating, but the host country as well. The extensive and most direct impact of IRM on the receiving countries has likely been the economic capital which the retired migrants have brought with them as they have bought or rented homes, provided employment for local workers, consumed goods and services, and attracted greater investments to the areas in which the retirees have resided [20,25,27]. The significant amount of revenue generated, particularly for some poorer areas, has likely had significant effects on receiving countries. For example, according to the Philippines Retirement Authorities’ estimation, the cross-border retirement industry yielded revenue of USD 2.4 billion in 2011 and was expected to increase yearly [9]. The influx of retired retirees has likely generated much needed jobs and promoted economic growth in the receiving countries; however, the drawbacks or negative effects on the receiving population would be rising real estate values and rental prices [25,26]. The inequality of spending power and wealth between the retired migrants and the locals resulted in some of the locals have been at risk of being displaced by higher prices [20,25,26,27]. Furthermore, the booming real estate business and land markets catered to the needs of the retired migrants have caused concern about ruining cultural authenticity of receiving countries, not to mention the demographic (e.g., the demographic trend would be toward an aging population), social-cultural (e.g., social-cultural integration of retired migrants and the local residents’ adaptation to this emerging trend of human mobility) and environmental implications (e.g., environmental damages and increased pollution) resulting from the trend of IRM [20,25,26,28]. Alternatively, IRM effects on sending countries, as more individuals have moved across national boundaries and have retired abroad, may have generated savings in the sending countries’ old-age support programs and had other consequential effects (Table 4) [29].

## 3. Discussion

Retirement abroad comes with both opportunities and challenges for retirees who embark on this journey. Retirees who report more optimal experiences are relatively younger, sociable, financially stable, and psychologically more patient and optimistic, with higher tolerance for frustration. Those retirees who engage in active lifestyles (e.g., socially, physically, and culturally) rate their experience more positively; this can occur by participating in social activities with fellow expatriates and local residents, volunteering in meaningful programs, teaching English, speaking the local language, taking financial investments, and maintaining financial, political and emotional ties with countries of origin. Additionally, the ability to accept the fact that life may not go as planned, while sustaining a realistic expectation of people (e.g., the local residents), place (e.g., the retirement location), and things (e.g., government bureaucracy), are essential in determining the retirees’ post-migration adjustment and satisfaction. Positive perceptions and cognitive resilience in older adults were found to be associated with optimal adjustment in later life [30,31]. On the other hand, the retired migrants have worst experiences when they are older, more introverted, lack family and social support, have not made time or engaged in wise financial management, are inflexible or impatient with the acculturation process and adjustment, are resistant to change, and have complicated medical issues or require long-term care accommodations. The older retired migrants with complex medical needs who do not speak the local language and lack family and social support are the most vulnerable and at-risk population to have poor IRM experiences. A language barrier may significantly impede access to healthcare and services, a situation which may compromise quality-of-life and be accompanied by feelings of loneliness, isolation, and even depression.

The retired migrants who steadfastly attempt to import their existing lifestyle from countries of origin into host countries often do not fare well when they encounter legal, medical, cultural, and governmental practices in host countries that differ from practices in the countries of origin. The retired migrants who are reserved, keep things to themselves, rarely participate in any social activities with others, and seldom reach out for help in difficult situations, tend to experience social isolation and failure in problem-solving. Retired migrants are often unable to endure the worldwide, ever-changing, social-economic-political landscape if they lack financial management skills or are unprepared for unexpected financial and political changes.

While retirees or soon-to-be retirees contemplate or make plans to retire abroad, they often experience hopes, dreams, expectations, anxiety, and other complex feelings. After retired migrants settle into host countries, acculturation stress with ongoing adjustment issues can be quite common. At this point, mental health professionals could play a vital role in guiding retirees or soon-to-be retirees toward making more informed decisions on IRM as well as in helping the retired migrants to make post-migration adjustment after they settle into host countries.

The results findings highlighted the complexities of IRM in which various levels (micro, meso and macro) of interconnected factors led to an individual’s eventual decision to choose retirement abroad rather than aging-in-place. A wide range of positive aspects of a foreign destination drove retirees from the high-income countries to move to and retire in low- or middle-income countries; these factors included the desire to live in a warmer climate with the possibility of engaging in a more active lifestyle, more affordable old-age care facilities/services, and a lower cost-of-living, all of which combined with prior travel/tourism experience and prior experience living abroad, facilitated the establishment of many incentives for retirement migration. Research findings confirmed that the approach of these cohorts of recent retirees to retirement and aging has been positive and innovative. Rather than passively accepting physiological decline, economic hardship, and narrowing social fields during retirement, retirees have been exhibiting a relatively greater degree of personal agency in the aging process and in the transition from full-time work to retirement by actively engaging in “successful” and “active” aging. The tendency toward actualization and sense of personal responsibility for post-retirement are in line with humanistic theory, which emphasizes an individual’s higher states of consciousness, self-actualization, growth, self-efficacy, and free will.

### 3.1. Opportunities

Aging is a natural but complex process involving a gradual, continuous accumulation of changes that encompasses declines in physical, psychological, and social functioning [32,33,34,35]. The ability to cope with aging is essential to an individual’s overall well-being, especially when one exits from the labor force and transition to a post-retirement phase with a new identity as retiree. Those retired migrants who have positive post-migration experience engaged in active and healthy lifestyle choices, such as by participating in outdoor activities, exploring the cultural and natural attractions which the country offered, social engagements with other expatriates or with the residents, and meaningful charities (e.g., volunteering, teaching English, etc.). Retiring to a warmer climate where an individual does not have to deal with extreme weather conditions (e.g., snow and ice) or other environmental exposures (e.g., air pollution) increased the likelihood of a physically active lifestyle and overall successful aging [36]. Living a healthy lifestyle was an important factor in improving regenerative capacity in neuronal synaptic and cognitive functioning, which eventually mitigated the aging process [37,38] and helped older adults optimizing functioning and quality of life as well as reducing morbidity and frailty as a person ages [34,35,39]. Social involvement provided individuals with a sense of structure and stability and also a sense of purpose and meaning [40]. As individuals moved and retired across international borders to a foreign destination, social and professional networks subsequently changed and evolved, as established connections were left behind. Thus, social participation in host countries provided the retired migrants an opportunity to reinvent themselves and grow personally while engaging in meaningful activities. This is aligned with humanistic theory which suggests that in order to be fully alive, individuals need to focus on the present moment, fully appreciate the present, and continuously to grow and to change. Therefore, as individuals of IRM utilized creative thinking and risk-taking ability to seek new challenges and experiences in life, goals focusing on adventure or self-growth provided ample opportunities to grow and to live a rewarding and fulfilling life.

### 3.2. Challenges 

While there were many benefits and opportunities in retiring abroad, there were many challenges as well. Moving abroad entailed more than a physical movement from one location to another. Relocation to a foreign destination also involved a psychological transition which affected nearly every aspect of the retired migrant’s life.

The most difficult challenge encountered by retired migrants was the language barrier. When retired migrants are unable to speak the local language, whether with local residents in a social conversation or with medical providers or nursing care staff in a medical setting, the communication barrier became a source of misunderstandings and mistrust between both parties; this obstacle not only prevented the retired migrants from getting whatever they needed, but further perpetuated unintended and unwanted isolation and loneliness. To overcome the language barrier, most retired migrants established closely-knit communities with fellow expatriates. While that situation can be helpful, it can also be detrimental in the acculturation process, as retirees isolated themselves from the local residents physically, socially, and culturally. This situation prevented them from fully integrating into the local community, which has a myriad of positive benefits associated with it.

As the retired migrant ages, there is a shifting of balance between ‘ways of being’ (the transnational practices migrants engaged in) and ‘ways of belonging’ (the identities and sense of self that resulted from connections to different locales) [41]. After a retired migrants’ spouses passes away or as friends in expatriate communities gradually died, the migrants were left without an existing social support network present in their lives. This resulted in a loss of identity, loneliness, and depression, which negatively affected well-being. These psychological aspects could be improved with mental health services, though if services were available, the retired migrants might not be able to utilize it if they were unable to communicate with the therapists using the local language. Untreated mental health issues made individuals susceptible to other comorbidities such as cardiovascular and metabolic diseases [42]. Therefore, the language barrier, combined with aging and retiring abroad where individuals lived far away from family and friends, significantly compromised the retired migrants’ quality of life—and this could be exacerbated by the inability to receive mental health services.

Other problems focused on poor medical care, political climate, and safety. Differences in medical and cultural practices between the retired migrants’ countries of origin and host countries alongside the quality of medical care were a main driving forces for retired migrants to repatriate to countries of origin. Political instability also made transnational retired migrants vulnerable in their pursuit of a better life; the magnitude and variety of the changes that have occurred in the world’s political systems created a challenge in retirees’ migratory experiences and experiences in daily life. Countries which used to be considered safe and were highly sought by retirees for an improved quality of life, may no longer hold the same reputations and be considered unsafe. Finally, economic challenges cannot be overlooked. As more international retirees moved into the host countries’ communities, there was a rise in the cost of rent, food, and services; retired migrants who desired to retire in a nice, pleasant, relaxed suburban area of the host countries found that the influx of international retirees also increased traffic congestion, housing and resort development, and construction of huge supermarkets or stores accommodating international retirees and tourists—in another words, the rising cost of living combined with air/environmental pollution, noise, and overcrowding disrupted the town’s peaceful and relaxing atmosphere.

After individuals make the decision to retire abroad and become settled into host countries, retired migrants may experience an initial period of anticipation and excitement. As early exhilaration and enthusiasm wear off, retired migrants should make necessary adjustments in order to adapt to the new culture and integrate into their host countries. The process of adjustment can be quite challenging and frustrating, especially when the retired migrants also have to deal with the process of aging. Therefore, mental health practitioners play a vital role in guiding the retired migrants to make post-migration adjustment after they settle into their host countries. With the development of telecommunication technologies, mental health services can be provided to individuals who have located in different continents and time zones via Skype, FaceTime or other video conferencing applications. Mental health assistance including Telehealth services are essential in helping retired migrants to build strategies in order to minimize the negative effects of aging and acculturation stress [43].

### 3.3. Recommendations for Research

The phenomenon of human migration has long been of research interest across multiple academic disciplines. Unfortunately, the emerging trend of IRM has not been extensively examined, either in academia or in public discourse. Due to deficiencies in available research, there is lack of empirical data to shed light on the new frontiers of transnational aging. Thus, there is a critical need to develop valid and reliable instruments, available in multiple languages, to assess and evaluate the retired migrants’ satisfaction and quality of life, post-retirement and post-migration. Additionally, future research should highlight: 1. social and culture integration of retired migrants; 2. the attitude of the local residents and communities in the receiving countries and adaptation to this emerging trend of human mobility; 3. the impact of IRM on the sending and receiving countries; and 4. the effects of returned migrants on countries of origin; 5. Gender dynamics, in terms of IRM and the ways older men and women deal with this phenomenon; and 6. the complexities of IRM and the Fourth Age older adults, in which individuals may experience grave biological and functional decline [44].

## 4. Conclusions

This study aimed to contribute to the understanding of the IRM phenomenon and to provide insight into the opportunities and challenges that international retired migrants encounter while retiring abroad. This study concluded that bio-psycho-social and financial factors were determinants in deciding the international retired migrants’ quality of life and their satisfaction post-retirement and post-migration. The retired migrants who were physically healthy, psychologically adaptive to changes, socially resourceful in terms establishing or utilizing social support network, and financially stable with wise investment and financial management strategies, were able to enjoy various opportunities for growth and fulfillment as a result of retiring abroad. Conversely, the retired migrants who have complex medical issues, were resistant to change, lack family and social support, were unable to speak the local language, and were financially unstable, risk having a more negative experience in retiring abroad.

More research is needed to gain a deeper understanding of the IRM phenomenon and its implications for, and impact on, the micro, meso, and macro levels of transnational aging. It is this writer’s hope that researchers in the fields of sociology, psychology, anthropology, and political science will undertake multidisciplinary research on IRM, in order to advance and expand the research literature available in this area, and to provide more insight into IRM through collective contributors.

## Figures and Tables

**Table 1:** International Retirement Migration Flow.

Source	Migration Flow		Article Type	Topic
	From	To		
Bender, Horn and Schweppe, 2017	Germans and Swiss	Thailand	Qualitative	Interview six old age care facilities
Benson and O’Reilly, 2018	Global North Retirees	Malaysia and Panama	Theoretical	Impact of IRM to the receiving countries
Botterill, 2016	Britain	Thailand	Qualitative	Interview 20 participants, between 50 to 70 years of age
Croucher, 2015	Global North	Global South	Theoretical	Impact of IRM to the receiving countries
Gambold, 2018	U.S	Mexico	Qualitative	Interview 78 participants between 2009–2010 and 2014–2015
	Britain	France and Spain		
Gehring, 2019	Dutch and Spanish	Dutch and Spanish	Qualitative	86 interviews of Dutch and Spanish retirement migrants moving or returning to Spain and Dutch and Dutch-Turkish retirement migrants moving or returning to Turkey after retirement
	Dutch-Turkish	Turkey		
Gustafson and Cardozo, 2017	Scandinavian	Spain	Qualitative	Interview 34 participants (14 Scandinavian retirees aged between 66 and 81 and 20 local residents)
Hall and Hardill, 2016	Britain	Spain	Qualitative	Interview 9 males and 16 females, averaged age was 78.25
Hamilton, 2015	Europeans, Americans, and Maltese	Malta	Qualitative	Interview 7 participants (2 males and 5 females aged between 51 and 75)
Hayes, 2018	N. Americans	Ecuador	Theoretical	Impact of IRM to the receiving countries
Horn and Schweppe, 2017	Global North	Global South	Theoretical	Transnational aging
Kline, 2013	Amenity migrants	Ecuador	Qualitative Thesis	Impact of IRM to the receiving countries
Kohno et al., 2016	Japan	Malaysia	Qualitative	Interview 38 participants (30 Japanese, 16 males and 14 females, aged from 54 to 79 years and 8 medical services providers)
Lardiés-Bosque, 2016	U.S.	Mexico	Qualitative	Interview 29 participants (15 males and 14 females, aged from 55 to 75 and older)
Marrow and von Koppenfels, 2018	U.S.	Global South	Theoretical	Migration aspirations
Matarrita-Cascante et al., 2017	Amenity/lifestyle migrants	Chile	Qualitative	Interview 46 participants (26 migrants and 22 local residents)
Miyashita et al., 2017	Japan	Thailand	Qualitative	Interview 237 participants (mean age 68.8, with 79.3% of them being male)
Rojas et al., 2014	U.S.	Mexico	Qualitative	Interview 375 participants (51.8 % of the subjects were male and 48.2% were female), averaged age was 68.05 years
Schafran and Monkkonen, 2011	U.S.	Mexico	Theoretical	Impact of IRM to the receiving countries
Toyota and Xiang, 2012	Japan	Thailand, Malaysia, and Indonesia	Theoretical	Interview 50 participants in Chiang Mai (Thailand), Penang (Malaysia), Cebu (the Philippines) and Bali (Indonesia)
Vega, 2015	Latin American	Retirees return to their birth countries from the U.S.	Quantitative	Quantitative method using a 1% sample of the Social Security Administration’s Master Beneficiary Record (MBR) and the Numerical Identification System database (NUMIDENT)
Wong, Musa, and Taha, 2017	European, American, Asian	Malaysia	Quantitative	Survey 504 participants (quantitative method, 64.3% of them aged 60 years and above)

**Table 2:** Pull Factors.

Source	Pull Factors Associated with International Migration
Economic	Lower cost of living
	Affordability of health care
	Affordability of housing
	Tax benefits
	Cheaper labor (domestic helping staff: maid, gardener, etc.)
	Investment opportunity (real estate, farming, retail business, etc.)
Destination	Pleasant climate, beautiful natural and cultural environment
	Urban amenities, such as advanced transportation infrastructures
	Easy access to recreation facilities for leisure, such as museums and parks
	Low crime rate
	Informal or relaxed lifestyle
	Same language spoken as the retired migrants’ country of origin
	Proximity to the retired migrants’ children and grandchildren, in order to be closer to family who may have already moved abroad
People	Well-established expatriate communities with like-minded retirees
	Friendly local residents
	Greater supply of skilled long-term care workers (e.g., Thailand, Malaysia, the Philippines)
Movement	Easy travel within the region (e.g., within EU countries, or between U.S. and Mexico)
	Easy accessibility to friends and families in their country of origin, due to increased global mobility in the transportation sector
	Simple-to-obtain visa and residency status

**Table 3:** Push Factors.

Source	Push Factors Associated with International Migration
Socio/Cultural Adjustment	Inability to adapt to the different culture and inability to integrate into the local community
	Different cultural expectations, and differences in understanding and mentality in care practice
	Lack of social support
	Host country was not what the retiree had expected it to be.
Financial Factors	Global economic downturn, unavailable retirement benefits
Healthcare Benefit	Medicare and SSI coverage
Political Risk	Unexpected or uncertain political changes, such as Brexit
Healthcare Approaches	Differences in medical systems and healthcare services between retiree’s country of origin and the host country. For example, the medical systems and healthcare services in Malaysia differ from those in Japan.

**Table 4:** Impact of International Retirement Migration on the Receiving Countries.

**Economic**	Even though the migrants helped job creation and promoted economic growth in the receiving countries, the rising real estate prices due to the influx of migrants may have placed some locals at risk of being displaced due to lack of affordability.
**Social**	Migrants’ purchasing power gave them the opportunities to be landowners, business owners, or employers. The locals became the employees of the migrants. Therefore, social classes were created, based on social and economic status, which widened the inequality between the migrants and the locals.
**Spatial**	Migrants resided in gated communities or apartment condos while many locals who were employed by the migrants resided in the impoverished area, which was segregated from where the migrants resided.
**Legal**	The receiving countries facilitated visas application, provided tax advantages, and relaxed rules for owning land and establishing business for the migrants, while the locals may not have had the same tax advantages as the migrants; therefore, the locals may have been put at a disadvantage in business competition.
**Environmental**	Environmental degradation caused by the new real estate development resulted in pollution, especially near the coastal area.
**Cultural**	Migration ruined the authenticity of the destination locations, including some UNESCO World Heritage sites.

## Data Availability

Not applicable.
